# The Cdc48 unfoldase prepares well-folded protein substrates for degradation by the 26S proteasome

**DOI:** 10.1038/s42003-019-0283-z

**Published:** 2019-01-21

**Authors:** Michal M. Olszewski, Cameron Williams, Ken C. Dong, Andreas Martin

**Affiliations:** 1 Department of Molecular and Cell Biology, University of California, Berkeley, CA 94720 USA; 2 California Institute for Quantitative Biosciences, University of California, Berkeley, CA 94720 USA; 3 Biophysics Graduate Group, University of California, Berkeley, CA 94720 USA; 4 Howard Hughes Medical Institute, University of California, Berkeley, CA 94720 USA

## Abstract

Cdc48/p97 is an essential and highly conserved AAA+ ATPase that uses its protein-unfoldase activity to extract ubiquitinated polypeptides from macromolecular complexes and membranes. This motor has also been implicated in protein-degradation pathways, yet its exact role in acting upstream of the 26S proteasome remains elusive. Ubiquitinated proteins destined for degradation by the proteasome require an unstructured initiation region to engage with the proteasomal translocation machinery, and Cdc48 was proposed to generate these unfolded segments, yet direct evidence has been missing. Here, we used an in vitro reconstituted system to demonstrate the collaboration of Cdc48 and the 26S proteasome from *S*. *cerevisiae* in degrading ubiquitinated, well-folded proteins that lack unstructured segments. Our data indicate that a critical role for Cdc48 in the ubiquitin-proteasome system is to create flexible initiation regions in compact substrates that otherwise would be refractory to engagement and degradation by the proteasome.

## Abstract (web summary)

Michal Olszewski et al. demonstrate the collaboration of Cdc48 and the 26S proteasome in degrading ubiquitinated, well-folded proteins that lack unstructured segments. This study suggests that Cdc48 creates flexible initiation regions in compact substrates for their proteasome-mediated degradation.

## Introduction

The ubiquitin proteasome system (UPS) maintains general protein homeostasis by degrading misfolded, aggregated, or folding-incompetent polypeptides, and controls numerous vital processes through the highly selective turnover of regulatory proteins^1^. In addition to the 26S proteasome as the major eukaryotic protease at the end of this pathway, the protein unfoldase Cdc48 (or p97/VCP in higher eukaryotes) has been implicated in ATP-dependent degradation^2^, yet how these molecular machines of the AAA+ (ATPases associated with diverse cellular activities) family collaborate within the ubiquitin-proteasome system remains largely unknown.

Degradation by the 26S proteasome is highly specific and tightly regulated through the labeling of appropriate substrates with lysine-attached ubiquitin chains and the sequestration of the proteasome’s proteolytic active sites within the internal degradation chamber of the barrel-shaped 20S core particle (CP)^3^. Access to these active sites is controlled by the 19S regulatory particle (RP), which is bound to one or both ends of CP and contains several ubiquitin receptors, the deubiquitinases Rpn11 and Ubp6/Usp14, and the ring-shaped, hetero-hexameric AAA+ motor of the proteasome. The RP recruits ubiquitinated substrates, engages them with conserved loops of its ATPase subunits in the central pore, and then uses ATP-hydrolysis-driven conformational changes for mechanical unfolding, deubiquitination, and translocation of the polypeptides into CP for proteolysis. Due to geometric constraints imposed by structures above the AAA+ motor, substrates must contain a flexible initiation region of at least 25–30 amino acids to reach and engage with the translocation machinery of the proteasome^4–7^. Even though a significant fraction of proteins in eukaryotic cells are predicted to include flexible regions of 30 residues or more^8^, it remained elusive how the proteasome can degrade the large number of proteins lacking intrinsically disordered regions.

Cdc48 is a highly conserved and essential homo-hexameric AAA+ ATPase required for a myriad of cellular processes^9,10^. It catalyzes the ubiquitin-dependent extractions of proteins from membranes and macromolecular complexes, and is assumed to function in the ubiquitin-proteasome system upstream of the proteasome, possibly in preparing substrates for degradation^11^. Furthermore, it has been proposed to functionally interact with the proteasomal CP to feed substrates directly into the degradation chamber for proteolysis^12–14^. Mutations in the human homolog p97 are linked to several neurodegenerative diseases^14,15^, and due to its general role in protein homeostasis it has been identified as a promising anti-cancer drug target^16,17^. The monomer consists of an N-terminal domain and two AAA+ domains termed D1 and D2 that in the hexamer assemble into stacked ATPase rings. Cdc48 recruits proteins with nonlinear ubiquitin chains through specific, N-domain bound cofactors, like the Ufd1/Npl4 (UN) heterodimer, and unfolds them by ATP-hydrolysis-dependent translocation through its central pore^18–20^.

Given its proposed role upstream of the 26S proteasome, we hypothesized that Cdc48 may be able to partially or completely unfold substrates that lack flexible segments and are therefore not directly susceptible to proteasomal degradation. Using an in vitro reconstituted system, we demonstrate the collaboration between these large AAA+ molecular machines in degrading compact, ubiquitinated proteins. Because Cdc48 does not depend on unstructured regions, it can play a key role in unraveling well-folded proteins for subsequent engagement and degradation by the 26S proteasome.

## Results

### Analysis of model-substrate degradation and unfolding

For a direct comparison, we aimed to characterize substrate unfolding by Cdc48 and Cdc48-mediated degradation by the 26S proteasome with largely identical proteins. We therefore created a model-substrate based on the photo-cleavable mEOS3.2 that allowed both unfolding and degradation to be monitored by fluorescence. For recognition by Ufd1/Npl4 and the ubiquitin receptors of the proteasome, mEOS3.2 was fused to a N-terminal linear tetra ubiquitin that was further modified with branched K48-linked chains by the Ufd2 ubiquitin ligase (Supplementary Figure 1A, B). The backbone of mEOS3.2 can be cleaved by UV irradiation, which leads to a shift in fluorescence emission from green to red^21^. Unfolding of the red protein variant by Cdc48 produces two fragments that are unable to refold, resulting in the irreversible loss of fluorescence. In contrast, the non-activated, green substrate readily refolds and regains its fluorescence after release from Cdc48, such that the permanent loss of the green fluorescence signal depends on degradation by the proteasome. These properties of mEOS3.2 thus allowed us to use the same preparation of the ubiquitinated substrate for multiple-turnover measurements of either degradation by the proteasome with the green, non-activated variant (K48-GREEN), or unfolding by Cdc48 using the red variant after photo-activation (K48-RED).

**Fig. 1:**
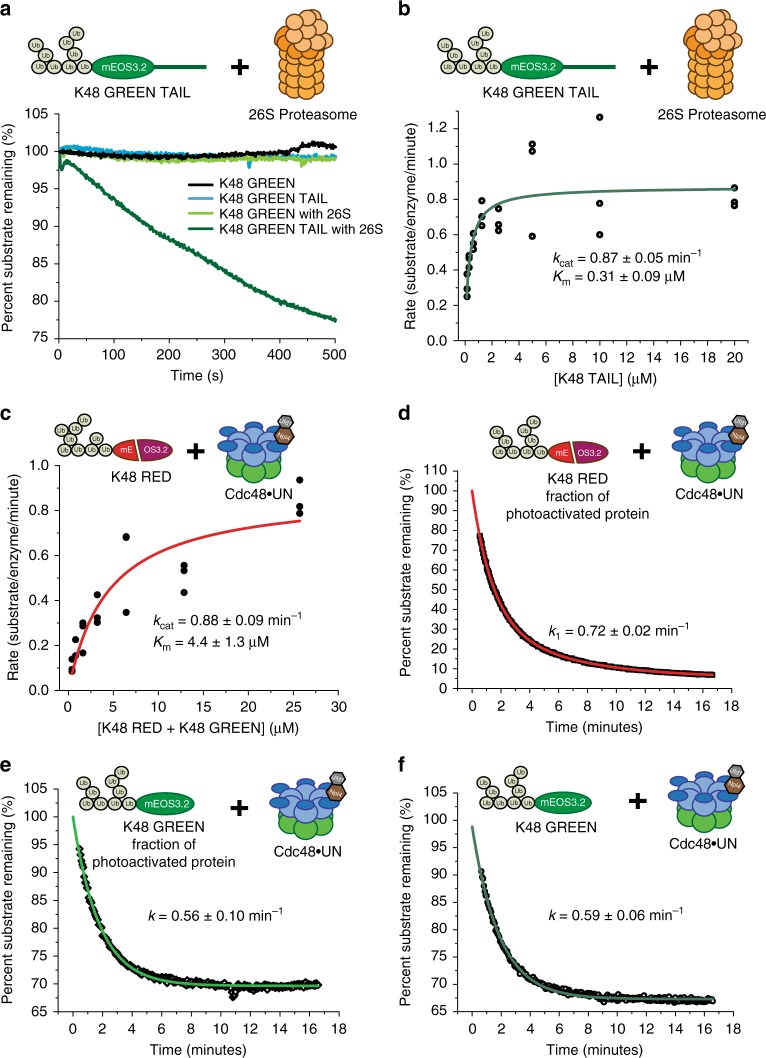
Quantitative analyses of model-substrate degradation by the proteasome and unfolding by Cdc48. **a** Representative fluorescence time courses of three separate experiments showing the proteasomal degradation of K48-GREEN TAIL and the lack of processing for tailless K48-GREEN. **b** Michaelis–Menten analysis of K48-GREEN TAIL degradation by the 26S proteasome. Shown are the individual rates for three repeats with identical protein samples and the fit. **c** Michaelis–Menten analysis of K48-RED unfolding by Cdc48 in the presence of Ufd1/Npl4 (Cdc48•UN). Shown are the individual rates for three repeats with identical protein samples and the fit. **d** Example fluorescence trace for the single-turnover unfolding of K48-RED by Cdc48•UN, fitted to a single exponential decay (red). **e** Example fluorescence trace for the single turnover unfolding of the K48-GREEN fraction of an activated K48-RED sample, fitted to single exponential decay (light green). **f** Example fluorescence trace for the single-turnover unfolding of non-activated K48-GREEN by Cdc48•UN, fitted to a single exponential decay (dark green). For **d–f**, rates were averaged from three measurements (*N* = 3, repeats with identical protein samples) with standard deviations reported. Missing points at the beginning of each measurement correspond to the dead time for manual mixing and sample transfer. **d** and **e** show processing of the same sample of activated mEOS3.2 substrate (red and green) monitored simultaneously at two different wavelengths. Raw data related to this figure is available in Supplementary Data 1

The ubiquitinated model substrate was designed to be very compact, without flexible segments or linkers that the 26S proteasome could engage (Supplementary Table 1). Consistently, we observed no proteasomal turnover of this substrate (Fig. 1a; Supplementary Figure 1C), which confirmed that degradation strictly requires the presence of an unstructured initiation region. However, a tailed version of this substrate (K48-GREEN-TAIL), containing a C-terminal 65 amino-acid extension derived from cyclin B^22^, was readily degraded with a *k*_cat_ of 0.87 min^−1^ and a *K*_M_ of 0.31 μM (Fig. 1b). This degradation did not depend on the presence of K48-linked ubiquitin branches, as the equivalent construct with only the linear tetra-ubiquitin fused to the N-terminus of mEOS3.2 (termed GREEN-TAIL) was also efficiently processed by the proteasome (Supplementary Figure 1C).

Unlike the 26S proteasome, Cdc48 can engage and unfold compact, tailless substrates. We observed robust unfolding of K48-RED in the presence of Ufd1/Npl4 (Cdc48•UN) under multiple-turnover conditions (Supplementary Figure 1D), for which Michaelis–Menten analyses revealed a *k*_cat_ of 0.88 min^−1^ and a *K*_M_ of 4.4 μM (Fig. 1c). No unfolding occurred in the absence of UN or when using the substrate variant that lacked K48-linked branches on the linear tetra-ubiquitin fusion (Supplementary Figure 1D). Previous studies of Cdc48 or p97 were limited to the analysis of single-turnover unfolding, because substrate translocation stalled on long ubiquitin chains^18^ and required ubiquitin cleavage by the deubiquitinase Otu1 for release from the complex^19^. In contrast, the branched and rather short ubiquitin modifications on our substrates allow processive, multiple-turnover unfolding by Cdc48. Considering previous observations of motor stalling on long ubiquitin chains, while substrate moieties themselves were completely threaded^18,19^, we favor a model for the processing of our substrates in which the short ubiquitin modifications are unfolded and co-translocated through Cdc48. That the central pore of Cdc48 has the plasticity to accommodate the multiple polypeptide chains that needed to be translocation for such a branched substrate is conceivable, given previous observations of other AAA+ motors, like the 26S proteasome and the bacterial ClpX, simultaneously translocating three or more overlapping polypeptides^23–25^.

Whereas measuring steady-state processing by Cdc48 required using the red substrate, which unfolds irreversibly, monitoring handoff to the proteasome depended on the refolding-competent green variant. To confirm that both forms are equivalently recognized and processed by Cdc48•UN, we directly compared their single-turnover unfolding. Photoactivation of mEOS3.2 leads to incomplete conversion^26^, and using a mixture with ~40% green and ~60% red substrate allowed us to simultaneously monitor unfolding of both variants within the same sample. The fluorescence changes for the red variant followed a double-exponential decay (Fig. 1d), in which the slower phase with lower amplitude probably originated from a differentially ubiquitinated species. The green substrate showed only single-exponential behavior (Fig. 1e), likely because the second, slow phase was masked by the increase in fluorescence when the fast-processed species reaches the steady-state equilibrium of Cdc48-induced unfolding and spontaneous refolding. Importantly, however, the dominant fast unfolding phase for K48-RED and K48-GREEN showed very similar rate constants of *k* = 0.72 min^−1^ and *k* = 0.56 min^−1^, respectively (Supplementary Table 2). Additionally, the kinetics for unfolding of the green fraction agreed with the rate for K48-GREEN that had not been exposed to photo-activation (*k* = 59 min^−1^, Fig. 1f). To confirm that the ubiquitinated green substrate is indeed fully translocated and not stalled on the complex, we performed a competition assay, in which we observed robust unfolding of K48-RED after Cdc48•UN had reached steady state in processing K48-GREEN (Supplementary Figure 2). Together, these data indicate that K48-GREEN is completely translocated and behaves like the red substrate in multiple-turnover unfolding by Cdc48.

### Cdc48 prepares substrates for ubiquitin-dependent degradation

**Fig. 2:**
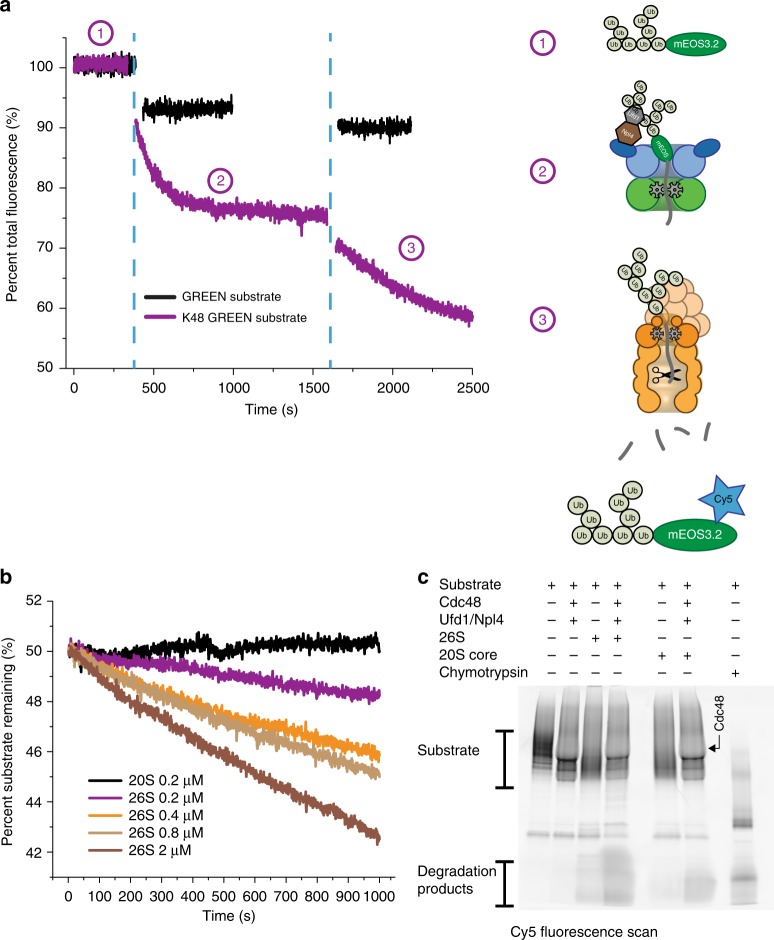
Cdc48-mediated substrate degradation by the 26S proteasome. **a** Fluorescence time course for the Cdc48-dependent proteasomal degradation of K48-GREEN (purple; shown is one example trace out of three experimental repeats). The substrate is equilibrated (1), before the addition of Cdc48•UN leads to unfolding (2). After reaching steady state of unfolding and refolding, 26S proteasome was added (3), and a continuous loss of fluorescence signal was observed, indicative of multiple-turnover degradation. No unfolding and therefore no degradation by the proteasome occurred when GREEN substrate without the branched ubiquitin chains was used (black trace). **b** The rate of K48-GREEN degradation after unfolding by Cdc48•UN correlates with the amount of proteasome added to the coupled reaction. **c** SDS-PAGE analysis of the endpoints for the Cdc48-coupled degradation assays of Cy5-labeled K48-GREEN. The gel was scanned for Cy5 fluorescence to detect peptide-product formation. Significant peptide formation depended on the presence of Cdc48•UN and the 26S proteasome. The apparent change in banding patterns upon addition of Cdc48•UN (lanes 2, 4, and 6) is likely caused by the large amounts of Cdc48 affecting the migration of ubiquitinated substrate in the gel. See Supplementary Figure 6 for image of entire gel. Raw data related to this figure are available in Supplementary Data 1

To test whether Cdc48 can prepare compact, well-folded proteins for proteasomal degradation by creating a flexible initiation region for engagement, we incubated K48-GREEN with Cdc48•UN, let the unfolding reaction reach steady state, and then added yeast 26S proteasome. We observed robust degradation as indicated by a continuous fluorescence decrease (Fig. 2a), whose amplitude correlated with the amount of proteasome in the reaction (Fig. 2b). The highest rate for this Cdc48-mediated degradation was *k*_deg_ = 0.135 min^−1^ proteasome^−1^, which is an order of magnitude lower than the maximal rate for the direct degradation of the tailed substrate variant under saturating conditions (*k*_ca_  =  0.87 min^−1^). This slow rate for K48-GREEN turnover likely originates from low concentrations of unfolded substrate available for proteasome engagement, caused by the limiting amount of Cdc48•UN in the reaction and the competing processes of substrate refolding or rebinding to Cdc48. Furthermore, the affinity of transiently unfolded substrate for the proteasome is expected to depend on the length, position, and identity of unstructured segments, as previous studies revealed the flexible initiation region as a major determinant for the *K*_M_ of proteasomal substrate processing^7,27^. Using larger amounts of Cdc48 in the reaction and thereby increasing the concentration of unfolded K48 GREEN for faster degradation was precluded by the high ATP-hydrolysis rate of Cdc48•UN (~ 970 min^−1^, Supplementary Figure 3) that led to rapid ATP depletion and inhibition of the proteasome, even in the presence of a strong ATP-regeneration system.

Importantly, however, the observed degradation clearly indicated the collaboration of Cdc48 and the proteasome holoenzyme in processing compact substrates. A substrate variant termed GREEN that lacked the branched ubiquitin chains and therefore was not unfolded by Cdc48 consequently showed no degradation by the proteasome (Fig. 2a). We also did not observe a decrease in K48-GREEN fluorescence when only Cdc48•UN and CP were present in the reaction (Supplementary Figure 4), even though it had been suggested that Cdc48 can interact with CP to function as an alternative protease complex^12,13^. Thus, Cdc48•UN and the 26S proteasome holoenzyme were required to catalyze the degradation of a well-folded substrate (Fig. 2b, Supplementary Figure 4).

To confirm that the observed loss of fluorescence was caused by proteasomal turnover, we monitored by SDS-PAGE the generation of peptide products during the degradation of K48 GREEN, whose surface-exposed cysteine was labeled with Cy5-maleimide. A sample containing substrate, Cdc48•UN, and the 26S proteasome showed robust peptide formation, whereas substrate incubation with only the proteasome or with Cdc48•UN and CP produced much lower amounts of peptide products (Fig. 2c). The slight degradation in the latter two samples may have originated from cleavage of partially unfolded, dark mEOS substrate, as we did not observe any degradation under the same conditions when monitoring green fluorescence (Figs. 1a, 2b). In addition to peptide-product formation, we also confirmed Cdc48-mediated proteasomal degradation by detecting Rpn11-catalyzed deubiquitination, which is known to be directly coupled to substrate translocation into the proteasome^23,28,29^. Monitoring the release of Cy5-modified ubiquitin revealed that robust deubiquitination of the K48-GREEN substrate by the proteasome required the presence of Cdc48•UN (Supplementary Figure 5). Proteasomes containing only the Rpn11 deubiquitinase thereby showed stronger substrate degradation and ubiquitin-chain release than proteasomes with Ubp6, whose non-catalytic inhibition of substrate processing has been previously described^30,31^. Our experiments thus confirm that Cdc48 prepares well-folded substrates for ubiquitin-dependent degradation by the 26S proteasome holoenzyme.

## Discussion

To our knowledge, we present the first direct evidence for the collaboration of Cdc48 and the 26S proteasome in degrading compact, well-folded proteins, where unraveling by Cdc48 generates flexible initiation regions that enable subsequent proteasomal processing. Our reconstitution identifies Cdc48•UN and the proteasome holoenzyme as the minimally required components for this coupled degradation, independent of shuttle receptors like Rad23 or the Otu1 deubiquitinase. The relatively short, branched ubiquitin chains on our model substrates appear to get unfolded and co-translocated by Cdc48, and may then refold more rapidly than the substrate itself, allowing ubiquitin-receptor binding while an unstructured region of the substrate polypeptide can engage with the ATPase motor of the proteasome. Interestingly, most poly-ubiquitin modifications on proteins in yeast soluble lysate have been found to include up to seven ubiquitin moieties^32^, which according to our and previous findings^19^ should allow complete translocation by Cdc48 without the need for deubiquitination by Otu1.

We designed the ubiquitinated substrate to lack any linkers or flexible termini, and it remains unclear where Cdc48 initiates unfolding. Recent structural studies suggested that the UN adaptor positions ubiquitin near the central pore of Cdc48, such that unfolding may start on a segment of the proximal ubiquitin moiety, which would constitute a universal initiation site for translocation irrespective of the attached substrate polypeptide^19^. We conclude based on our findings that initial unraveling must be facilitated by the D1 domains and/or N-terminal domains of Cdc48. Unfolding and threading solely by the D2 domains, as previously proposed, would require the substrate to contain a flexible initiation region of similar length as the ones for proteasomal engagement, which is clearly not the case.

After complete unfolding, the substrate is likely released by Cdc48 and diffuses to the 26S proteasome, but future studies can address whether any direct interaction between these molecular machines occurs for substrate handover. While competing reactions under our experimental conditions prevented measuring the maximal velocity of Cdc48-coupled proteasomal degradation, substrate transfer in vivo is expected to be facilitated by interactions with the shuttle receptors Rad23 and Dsk2, which may stabilize the unfolded state of substrates and increase their affinity for the proteasome^33–35^. Furthermore, the Cdc48-bound deubiquitinase Otu1 and the E4 ligase Ufd2 may edit and extend substrate-attached ubiquitin modifications at Cdc48 to promote binding to the proteasome and restrain competing rebinding to adaptors for Cdc48.

Cdc48 is involved in a myriad of processes, and not all of its unfolding activity leads to degradation. Our reconstituted system enables important future investigations into how Cdc48 and the proteasome decide the fate of a protein, how different ubiquitin modifications are recognized by Cdc48 adaptors and the proteasome, and how these modifications may be edited by Otu1 and Ufd2 to modulate substrate processing and determine the order of interactions with these molecular machines.

## Methods

### Expression and purification of Cdc48, Ufd1, Npl4, and Ufd2

The full-length sequence of Cdc48 was cloned into the pETDuet vector using BamHI and HindIII sites, with an N-terminal PreScission protease site. Ufd1 was cloned into the pET 24(+) vector using NdeI and XhoI sites, with a C-terminal PreScission site placed before the His_6_ tag. Npl4 was cloned into the pETDuet vector using EcoRV and XhoI. Ufd2 was cloned into pETDuet using EcoRI and SalI sites with an N-terminal PreScission protease sequence.

BL21* (DE3) cells were transformed with the expression plasmids for Cdc48 and its cofactors as well as the pACYC RIL vector for constitutive expression of rare tRNAs. Ufd1 and Npl4 were co-expressed and purified together. Cells were grown in Terrific Broth media supplemented with 1% glycerol at 37 °C to OD_600_ = 1.0−1.2. Protein expression was induced overnight at 18 °C with 1 mM isopropyl β-D-thiogalactopyranoside (IPTG). Cells were harvested by centrifugation at 6000×*g* for 25 min and resuspended in lysis buffer 1 (60 mM HEPES, pH 7.6, 200 mM NaCl, 0.5 mM EDTA, 10% glycerol and 30 mM imidazole). The lysis buffer for Cdc48 (lysis buffer 2) additionally contained 10 mM MgCl_2_. Lysis buffers were supplemented with 2 mg/mL lysozyme, benzonase (Novagen), and protease inhibitors (aprotinin, pepstatin, leupeptin, and PMSF).

All purification steps were performed at 4 °C. Cells were lysed by freeze-thaw and sonication. The lysates were clarified by centrifugation at 15,000 rpm for 30 minutes and the soluble extracts were bound to Ni-NTA agarose resin (Qiagen) and washed with lysis buffer 1 for cofactors, and buffer 2 supplemented with 1 mM ATP for Cdc48. The bound cofactor proteins were eluted with buffer 1 plus 300 mM imidazole. The bound Cdc48 was eluted with buffer 2 plus 1 mM ATP and 300 mM imidazole. The eluate was treated with PreScission protease overnight at 4 °C and dialysed into buffer 4 (60 mM HEPES, pH 7.6, 200 mM NaCl). Cdc48 was treated with PreScission protease overnight at 4 °C, and was buffer exchanged using spin concentrators into buffer 5 (60 mM HEPES, pH 7.6, 200 mM NaCl, 10 mM MgCl_2_, 1 mM ATP). Protein solutions were then passed over fresh Ni-NTA resin to remove the PreScission protease and uncleaved protein. The flow-through was collected and concentrated using Amicon Ultra spin concentrator (Millipore) with appropriate MW cut-offs. Concentrated proteins were filtered through a 0.22 μm spin filter and subjected to size-exclusion chromatography. Cdc48 was run over a Superose 6 column 16/70 (GE) pre-equilibrated with gel filtration buffer 1 (60 mM HEPES, pH 7.6, 200 mM NaCl, 10 mM MgCl_2_, 1 mM ATP, 5% glycerol), whereas Ufd2 and the Ufd1/Npl4 heterodimer were purified using Superdex 200 16/60 column (GE) and gel filtration buffer 2 (60 mM HEPES, pH 7.6, 200 mM NaCl, 5% glycerol). Peak fractions were concentrated and the protein concentration determined by absorbance at 280 nm. Due to the presence of ATP, the concentration of Cdc48 was determined by Bradford assay (Biorad). Concentrated proteins were aliquoted, frozen in liquid nitrogen, and stored at −80 °C.

### Expression and purification of ubiquitin

Ubiquitin was expressed and purified as described^36,37^. Briefly, *E. coli* Rosetta II (DE3) pLysS cells were transformed with a pET28a vector containing the ubiquitin gene from *S. cerevisiae* under control of a T7 promoter. Cells were grown in Terrific Broth supplemented with 1% glycerol at 37 °C until OD_600_ = 1.5–2.0 and were induced with 0.5 mM IPTG overnight at 18 °C. Cells were harvested by centrifugation and resuspended in ubiquitin lysis buffer (50 mM Tris-HCl, pH 7.6, 0.02 % NP-40, 2 mg/mL lysozyme) with added benzonase (Novagen) and protease inhibitors (aprotinin, pepstatin, leupeptin, and PMSF). Cells were lysed by sonication and 20 min incubation at room temperature. Lysate was clarified by centrifugation at 15,000 rpm for 30 minutes and the soluble extract was precipitated by addition of 60% perchloric acid to a final concentration of 0.5%. The solution was stirred on ice for 20 min. A 5 mL HiTrap SP FF column (GE Life Sciences) was used for cation-exchange chromatography, and ubiquitin fractions were pooled and run over a Superdex 75 16/60 (GE) in ubiquitin-storage buffer (20 mM Tris-HCl, pH 7.6, and 150 mM NaCl).

### Expression and purification of E1 and E2 ubiquitin ligases

E1 Uba1 and E2 Ubc4 were expressed and purified as previously described^36,38^. Briefly, Uba1 and Ubc4 were expressed in BL21 (DE3) in Terrific Broth at 37 °C until OD_600_ = 0.6–1.0 and induced with 1 mM IPTG overnight at 18 °C. Cells were harvested by centrifugation and resuspended in buffer A (60 mM HEPES 7.6, 250 mM NaCl with lysozyme, benzonase (Novagen), and protease inhibitors (aprotinin pepstatin, and PMSF). Cells were lysed by sonication and clarified by centrifugation at 15,000 rpm for 45 minutes and batch purified using Ni-NTA resin (Thermo). Resin was washed with buffer A+20 mM imidazole and protein was eluted with buffer A+250 mM imidazole. E1 was further purified over a Superdex 200 16/60 (GE) using storage buffer (60 mM HEPES 7.6, 200 mM NaCl, and 10% glycerol), while E2 was purified over a Superdex S75 16/60 (GE) in the same storage buffer. Samples were aliquoted and flash frozen in liquid nitrogen.

### Expression and purification of SENP2

SENP2 was expressed from pET28a-SENP2 (catalytic domain), a gift from Guy Salvesen (Addgene plasmid # 16357), and purified as previously described^7^. Briefly, *E. coli* BL21 star(DE3) transformed with pET28a-SENP2 were grown in Terrific Broth at 37 °C until OD_600_ = 0.6–1.0 and induced with 1 mM IPTG overnight at 18 °C. Cells were harvested by centrifugation and resuspended in buffer A (60 mM HEPES 7.6, 250 mM NaCl with lysozyme, benzonase (Novagen), and protease inhibitors (aprotinin pepstatin, and PMSF). Cells were lysed by sonication, the lysate was clarified by centrifugation at 15,000 rpm for 45 minutes, and the SENP2 was purified using HisTrap column (Thermo), followed by size-exclusion chromatography on a Superose 75 16/60 column equilibrated in storage buffer (60 mM HEPES 7.6, 200 mM NaCl, and 10% glycerol).

### Expression and purification of substrates

The construct for GREEN substrate was created by sub-cloning a sequence of mEOS3.2 with C-terminal intein—Chitin Binding Domain tag using Gibson cloning into a pET15b vector containing His_6_-SUMO-tetra ubiquitin (Gensript). The GREEN-TAIL substrate was generated by C-terminal fusion of a 65 amino-acid sequence derived from cyclinB and containing ssrA tag (AANDENYALAAHGGKHTFNNENVSARLGGACSIAVQAPAQHTFNNENVSARLGGALSIAVQAPAQ)^22^ before the intein. Both the GREEN and GREEN-TAIL substrates were expressed and purified in the same way, as described below.

BL21* (DE3) cells containing the respective expression vector were grown in Terrific Broth media supplemented with 1% glycerol at 30 °C to OD_600_ = 0.8–1.0, and protein expression was induced overnight at 18 °C with 1 mM IPTG. Cells were harvested by centrifugation at 6000×*g* for 25 min and resuspended in lysis buffer 1. Cells were lysed and proteins purified using Ni-NTA agarose resin the same way as for the Cdc48 cofactors. Following elution, substrate proteins were dialyzed into buffer 4 and cleaved overnight at 4 °C with SENP2 protease to remove the N-terminal His6-SUMO tag. Subtractive IMAC was used to remove His_6_ tagged SENP2 and uncleaved substrate proteins. The flow-through containing cleaved substrate was incubated with Chitin Resin (NEB), and bound substrate was eluted using buffer 4 with 50 mM DTT at 4 °C overnight. Eluted substrate was concentrated using a spin filter (MWCO 30,000 Da), filtered through a 0.22 μm spin filter, and further purified using a Superdex S200 16/60 pre-equilibrated in buffer 4 with 1 mM DTT. Peak fractions were concentrated, quantified using absorbance at 507 nm, aliquoted, and frozen in liquid nitrogen to be stored at −80 °C.

### Activation of K48 GREEN substrate

Following ubiquitination and purification, a fraction of K48 GREEN was photoactivated using a 405-nm laser. The activation consisted of ten 2-min on and off cycles, with the laser set to 50 mW.

### Purification of yeast 26S proteasome, yeast 20S core particle, and yeast 19S regulatory particle

The 26S proteasome and 19S RP were purified from strain YYS40 (genotype *MAT*a *ade2-1 his3-11,15 leu2-3,112 trp1-1 ura3-1 can1 Rpn11::Rpn11-*3× Flag)^39^ as previously described^31,40^. The 20S CP was purified from strain yAM14 (genotype *MAT*a *ade2-1 his3-11,15 leu2-3,112 trp1-1 ura3-1 can1-100 bar1 PRE1::PRE1-*3× Flag*(KanMX*)^22^, as previously described^31,41^. The 26S proteasome lacking Ubp6 deubiquitinase was purified from YYS40 strain (MATa ade2-1 his3-11,15 leu2-3,112 trp1-1 ura3-1 can1-100 RPN11::RPN11-3XFLAG (HIS3) ubp6∆::KANMX6)^31,41^. Yeast cultures for the 26S holoenzyme, ∆Ubp6 26S, 19S RP, and 20S core preparations were grown in YPD media for 3 days or until saturation. Cells were pelleted, weighed, and resuspended in buffers. HE lysis buffer consisted of 60 mM HEPES, pH 7.6, 75 mM NaCl, 75 mM KCl, 10 mM MgCl_2_, 0.5 mM EDTA, 5% glycerol, and 0.2% NP-40 with 1x ATP Regeneration mix (5 mM ATP, 0.03 mg/ml creatine kinase and 16 mM creatine phosphate). Lysis buffer for the 19S regulatory-particle preparation contained 60 mM HEPES, pH 7.6, 500 mM NaCl, 10 mM MgCl_2_, 0.5 mM EDTA, 5% glycerol, and 0.2% NP-40 with 1x ATP Regeneration mix (5 mM ATP, 0.03 mg/ml creatine kinase and 16 mM creatine phosphate). Core lysis buffer contained 60 mM HEPES, pH 7.6, 500 mM NaCl, 100 mM KCl, 0.5 mM EDTA, 5% glycerol, and 0.2% NP-40.

Cells were lysed by cryo-grinding using a Freezer Mill (Spex Sample Prep), and the cell powder was thawed at 25 °C. Lysate was cleared by centrifugation and the soluble fraction was batch bound to M2 anti-FLAG resin (Sigma). Resin with bound holoenzyme or ∆Ubp6 26S was washed with HE wash buffer (60 mM HEPES, pH 7.6, 75 mM NaCl, 75 mM KCl, 10 mM MgCl_2_, 5% glycerol, 0.1% NP-40 and 500 mM ATP). Bound 19S RP was washed with 60 mM HEPES, pH 7.6, 500 mM NaCl, 10 mM MgCl_2_, 5% glycerol, 0.1% NP-40, and 500 mM ATP. Core wash buffer was composed of 60 mM HEPES, pH 7.6, 500 mM NaCl, 75 mM KCl, 5% glycerol, 0.1% NP-40. Proteins were eluted with 0.15 mg/mL 3X Flag peptide and ran on a Sup6i 10/300 GL (GE) column pre-equilibrated with yeast gel filtration buffer (60 mM HEPES, pH 7.6, 75 mM NaCl, 75 mM KCl, 5% glycerol) for core and with proteasome gel filtration buffer for holoenzyme and ∆Ubp6 26S (60 mM HEPES, pH 7.6, 75 mM NaCl, 75 mM KCl, 10 mM MgCl_2,_ 500 mM ATP, 1 mM DTT, 5% glycerol). 19S RP gel filtration buffer contained 60 mM HEPES, pH 7.6, 75 mM NaCl, 75 mM KCl, 10 mM MgCl_2,_ 500 mM ATP, 0.5 mM TCEP, and 5% glycerol. Pooled fractions were concentrated, and frozen in liquid nitrogen. Protein concentration was determined via absorbance at 280 nm for 20S core and Bradford assay for 26S holoenzyme, ∆Ubp6 26S, and 19S RP.

### Purification of Ubp6 protein

Ubp6 was expressed and purified as previously described^31^. Briefly, BL21* (DE3) cells were grown in Terrific Broth media supplemented with 1% glycerol at 37 °C to OD_600_ = 0.6, and protein expression was induced overnight at 18 °C with 0.5 mM IPTG. Cells were harvested by centrifugation at 6000×*g* for 25 min and resuspended in lysis buffer 1. Cells were lysed and protein purified using Ni-NTA agarose resin, followed by size-exclusion chromatography using a Superdex-S200 column (GE).

### Ubiquitination of substrates

mEOS3.2 substrates with N-terminal tetra-ubiquitin fusion at a final concentration of 120 μM was premixed with Uba1 (8 μM), Ubc4 (30 μM), Ufd2 (36 μM), and 1.6 mM ubiquitin. All enzymes contained His_6_ tag, whereas the substrates were tag-less. The ubiquitination reaction was initiated with ATP (2 mM), incubated at 25 °C for three hours, diluted in buffer 1, and subjected to subtractive IMAC (1 ml HisTrap HP column (GE)) to remove all enzymes. The flow-through was concentrated, filtered through a 0.22 μm spin filter, and injected onto Sup6i 10/300 GL (GE) column pre-equilibrated with gel filtration buffer 2, to separate ubiquitinated substrate from free ubiquitin chains. Peak fractions were concentrated, quantified using absorbance at 507 nm, aliquoted, and frozen in liquid nitrogen to be stored at −80 °C.

### Multiple- and single-turnover unfolding assays

Unfolding assays were measured by monitoring the loss of fluorescence for K48-GREEN (excitation 500 nm, emission 520 nm) and K48-RED (excitation 565 nm, emission 585 nm) using QuantaMaster spectrofluorometer (PTI) at 30 °C.

For single turnover assays, substrate K48-GREEN or K48-RED at a final concentration of 0.2 μM was first preincubated with 100 μM creatine phosphate, Ufd1/Npl4 heterodimer (17 μM final), and 4X ATP Regeneration Mix. The sample was then diluted by adding an equal volume of Cdc48 (17 μM final), BSA (0.5 mg/ml final), 100 μM creatine phosphate and 1X ATP Regeneration Mix. Single-turnover curves were fit to single exponential (K48-GREEN) and double-exponential decay (K48-RED) using Origin Software (Originlab, Northampton, MA).

For multiple-turnover assays, K48-RED substrate at 20 μM was first preincubated with Ufd1/Npl4 heterodimer (15 μM final) and creatine phosphate (100 μM final), before adding an equal volume of Cdc48 (0.2 μM final) with BSA (0.5 mg/ml final) and 1X ATP Regeneration Mix (final concentration). For Michaelis–Menten analyses, substrate concentrations were varied from 0.4 μM to 26 μM, with each condition repeated in triplicate. Calculated initial rates were fit directly to the Michaelis–Menten equation with nonlinear regression using Origin Software (Originlab, Northampton, MA).

### Degradation of K48-GREEN-TAIL substrate by the 26S proteasome

Degradation assays were measured by monitoring the loss of fluorescence of K48-GREEN-TAIL (excitation 500 nm, emission 520 nm) using a QuantaMaster spectrofluorometer (PTI) at 30 °C. For multiple-turnover degradation assays, the K48-GREEN-TAIL substrate at 20 μM was preincubated with BSA (0.5 mg/ml final), and creatine phosphate (100 μM final), before adding an equal volume of 26S holoenzyme solution (0.4 μM final) with 1X ATP Regeneration Mix (final concentration) in reaction buffer. Michaelis–Menten analyses were performed with 0.2 μM 26S proteasome and substrate concentrations varied between 0.27 μM and 35 μM. Calculated initial rates were fit to the Michaelis–Menten equation with nonlinear regression using Origin Software (Originlab, Northampton, MA).

### Cdc48-dependent proteasomal degradation of K48-GREEN and GREEN substrates

Degradation was measured by monitoring the loss of green mEOS fluorescence (excitation 500 nm, emission 520 nm), using a QuantaMaster spectrofluorometer (PTI) at 30 °C. K48-GREEN or GREEN substrate (20 μM final) were preincubated with BSA (0.5 mg/ml final), Ufd1/Npl4 (15 μM final), Cdc48 (2 μM final), and creatine phosphate (100 μM final). No unfolding was observed until initiation by the addition of ATP Regeneration Mix (2x final concentration). After ~15 minutes, when the reaction had reached steady state, the 26S proteasome was added together with more ATP Regeneration Mix (2x final concentration). 26S proteasome concentrations were varied between 0.2 μM and 2 μM final. 20S CP at 0.2 μM final was added instead of the 26S holoenzyme in a subset of reactions. For experiments with reconstituted 26S proteasome, 20S CP and 19S RP were premixed at final concentrations of 0.4 μM and 1.6 μM, respectively. For experiment presented in Fig. 2a, the protein concentrations were as follows, Cdc48 0.2 μM, K48-GREEN or GREEN substrate at 2 μM and 26S proteasome at 0.2 μM.

### SDS-PAGE analysis of Cdc48-dependent proteasomal degradation

GREEN substrate was modified on Cys195 with Cy5-maleimide, separated from free dye using a Superose 6 column 16/70 (GE), and subjected to ubiquitination using the same conditions as for GREEN substrate described above. K48-GREEN-Cy5 was further purified in the same way as K48-GREEN. For Cdc48-coupled degradation, 18 μM substrate K48-GREEN-Cy5, 18 μM Ufd1/Npl4, 3 μM Cdc48 and 0.4 μM of either 26S proteasome or 20S CP were used. Reactions also contained 2x ATP Regeneration Mix, 0.5 mg/ml BSA and 50 μM creatine phosphate. Reactions were incubated for 30 minutes at 30 °C, quenched with SDS, and analyzed by SDS-PAGE. As a positive control for peptide formation, K48-GREEN-Cy5 was incubated with chymotrypsin.

### SDS-PAGE analysis of Cdc48-dependent substrate deubiquitination during proteasomal degradation

GREEN and GREEN-TAIL substrates were modified with Cy5-labeled ubiquitin (Cy5-K48-GREEN and Cy5-K48-GREEN-TAIL). Ubiquitinated proteins were purified as described above and subjected to Cdc48-dependent degradation, using 18 μM substrate, 3 μM Ufd1/Npl4, 3 μM Cdc48, 0.4 μM of either 20S CP or ΔUbp6 proteasomes in the absence or presence of 0.4 μM recombinant Ubp6, a 2x ATP Regeneration Mix, 0.5 mg/ml BSA, and 50 μM creatine phosphate. For control reactions, deubiquitination by Rpn11 and Ubp6 was inhibited with 3 mM ortho-phenanthroline (OPA) and 3 μM ubiquitin-vinyl sulfone. Reactions were incubated for 30 minutes at 30 °C, quenched with SDS, and analyzed by SDS-PAGE to detect cleaved-off, Cy5-labeled ubiquitins.

### ATPase assay

ATP hydrolysis rates were determined using a spectrophotometric assay that couples ADP formation to the depletion of NADH^42^. Cdc48 (with or without Ufd1/Npl4) and ATPase mix were incubated separately at 30 °C for 20 min and then combined in a preheated 96-well plate (Greiner). Absorbance at 340 nm was monitored in 9 sec intervals for 25 min with a Molecular Devices SpectraMax 190 microplate reader. Cdc48 and Ufd1/Npl4 were present at final concentrations of 50 nM and 2 μM, respectively. Reactions were performed in 1X ATPase mix (5 mM ATP, 3 U mL^−1^ pyruvate kinase (Sigma), 3 U mL^−1^ lactate dehydrogenase (Sigma), 1 mM NADH, 7.5 mM phosphoenol pyruvate (Sigma)) and buffer (60 mM HEPES, pH 7.6, 200 mM NaCl, 10 mM MgCl_2_, 5% glycerol). ATPase rates were extracted from the linear region of the absorbance trace using the extinction coefficient for NADH (6.22 mM^−1^ cm^−1^).

## Supplementary information

Supplementary Information

Supplementary Data 1

Description of Additional Supplementary Files

## Data Availability

The authors declare that all data supporting the findings of this study are available within the paper and its supplementary information. Please contact a.martin@berkeley.edu for plasmids.
