# Supplementary material for: The Cdc48 unfoldase prepares well-folded protein substrates for degradation by the 26S proteasome
Source: Commun Biol. 2019 Jan 21;2:29. doi: 10.1038/s42003-019-0283-z (PMC6340886; doi:10.1038/s42003-019-0283-z)
Supplement: Supplementary file 1 — Supplementary Information [file 42003_2019_283_MOESM1_ESM.pdf]

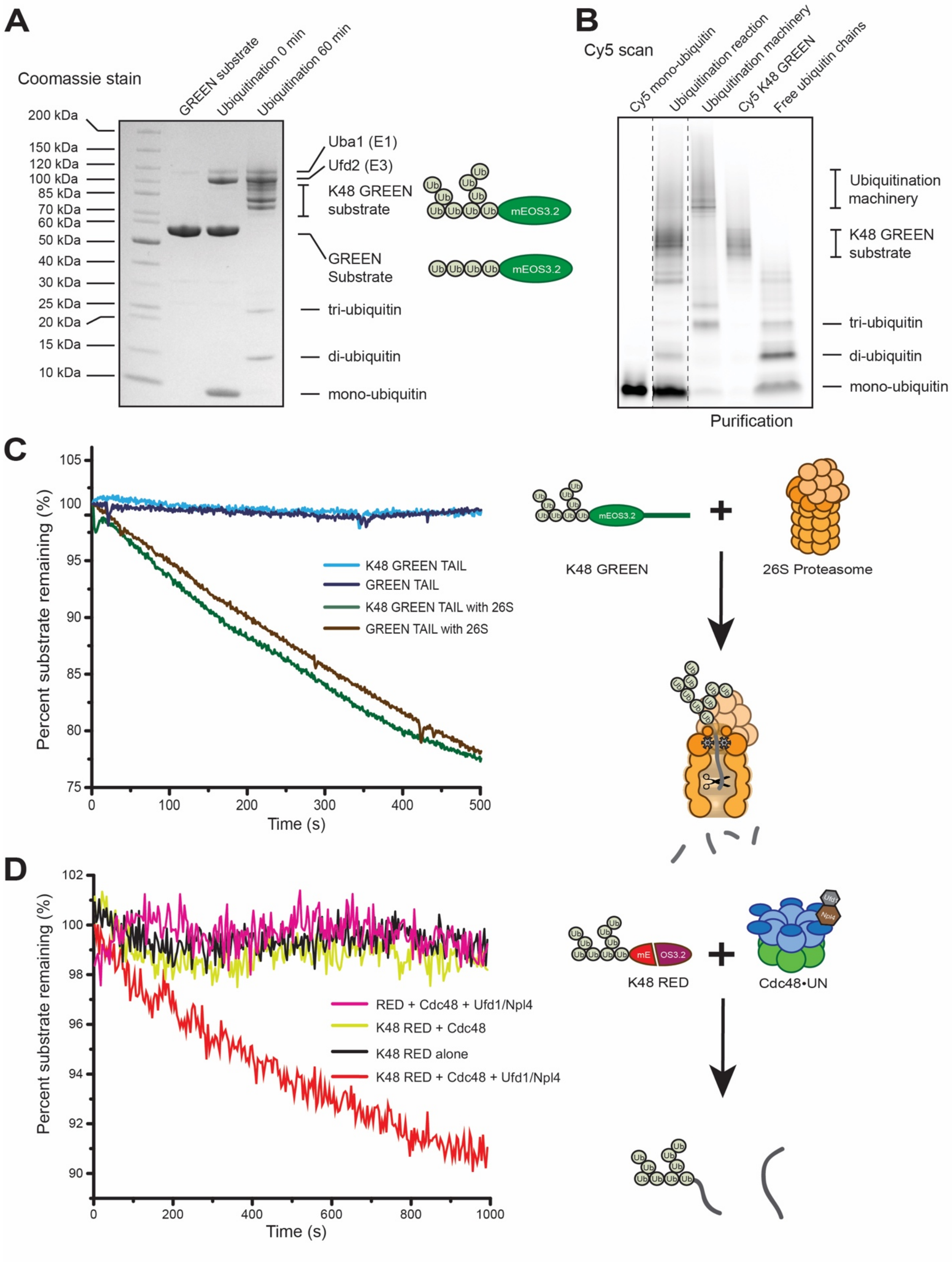
A
Coomassie stain
GREEN substrate
Ubiquitination 0 min
Ubiquitination 60 min
200 kDa
150 kDa
120 kDa
100 kDa
85 kDa
70 kDa
60 kDa
50 kDa
40 kDa
30 kDa
25 kDa
20 kDa
15 kDa
10 kDa
Uba1 (E1)
Ufd2 (E3)
K48 GREEN substrate
GREEN Substrate
tri-ubiquitin
di-ubiquitin
mono-ubiquitin
Ub
mEOS3.2
B
Cy5 scan
Cy5 mono-ubiquitin
Ubiquitination reaction
Ubiquitination machinery
Cy5 K48 GREEN
Free ubiquitin chains
Ubiquitination machinery
K48 GREEN substrate
tri-ubiquitin
di-ubiquitin
mono-ubiquitin
Purification
C
Percent substrate remaining (%)
Time (s)
K48 GREEN TAIL
GREEN TAIL
K48 GREEN TAIL with 26S
GREEN TAIL with 26S
K48 GREEN
26S Proteasome
D
Percent substrate remaining (%)
Time (s)
RED + Cdc48 + Ufd1/Npl4
K48 RED + Cdc48
K48 RED alone
K48 RED + Cdc48 + Ufd1/Npl4
mE
OS3.2
K48 RED
Cdc48•UN
Ufd1
Npl4

Supplementary Figure 1. Substrate ubiquitination, and qualitative analyses of proteasomal degradation and unfolding by Cdc48. (A) Ubiquitination leads to the depletion of GREEN substrate and mono-ubiquitin, and the appearance of bands for modified K48-GREEN substrate at higher molecular weights, indicative of successful ubiquitination. **(B)** Cy5-fluorescence scanned SDS-PAGE gel, showing the modification of K48-GREEN substrate with Cy5-labeled ubiquitin and its purification by affinity and size-exclusion chromatography. The His_6-tagged ubiquitination machinery was removed by immobilization on Ni-NTA (eluate shown in lane 3), and the flow-through was further purified by size-exclusion chromatography to separate the ubiquitinated substrate (lane 4) from free ubiquitin species (mono-ubiquitin and unanchored chains, lane 5). The K48-GREEN-TAIL substrate was generated in the same way (data not shown) **(C)** Representative fluorescence time courses for the degradation of K48-GREEN-TAIL and GREEN-TAIL by the 26S proteasome, which show similar kinetics. **(D)** Multiple-turnover unfolding of K48-RED by Cdc48 in the presence of Ufd1/Npl4 (Cdc48•UN). Unfolding is dependent on the UN cofactor and substrate modification with branched K48-linked chains.

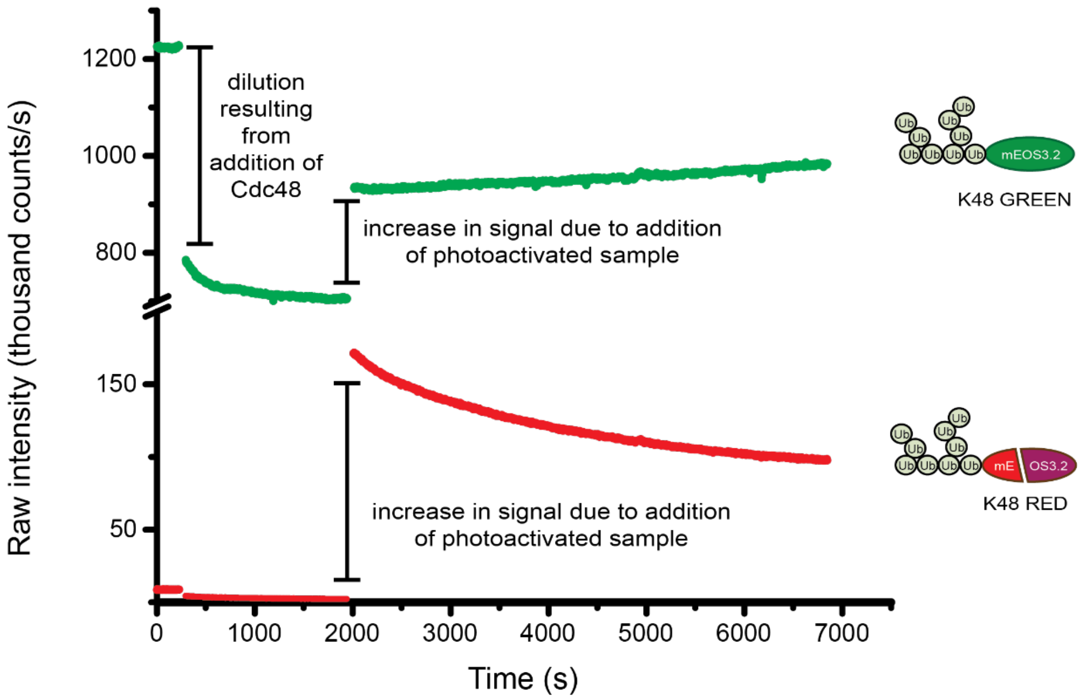

Supplementary Figure 2. Unfolding-competition assay reveals complete translocation and release of K48-GREEN by Cdc48. An excess of K48-GREEN substrate (20 µM) was subjected to unfolding by Cdc48•UN (2 µM). After reaching steady-state unfolding, photoactivated sample composed of ~ 12 µM K48-RED and ~ 8 µM K48-GREEN was added, and unfolding of the RED fraction as well as refolding of the GREEN fraction was observed.

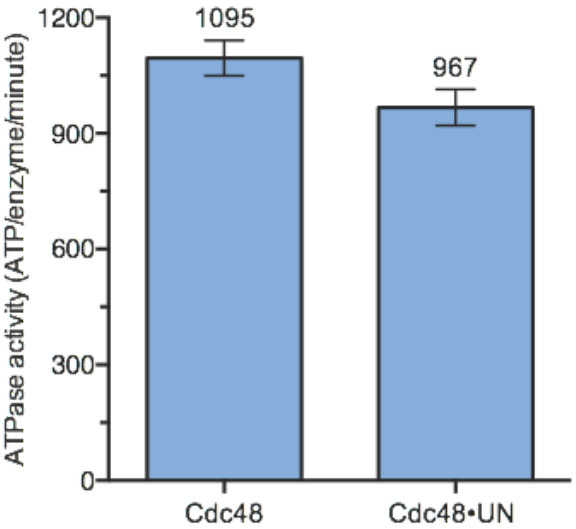

Supplementary Figure 3. ATP hydrolysis rates of Cdc48 and Cdc48•UN. ATP hydrolysis rates were determined for Cdc48 alone and in the presence of Ufd1/Npl4 (Cdc48•UN). Shown are the means and standard deviations of three measurements.

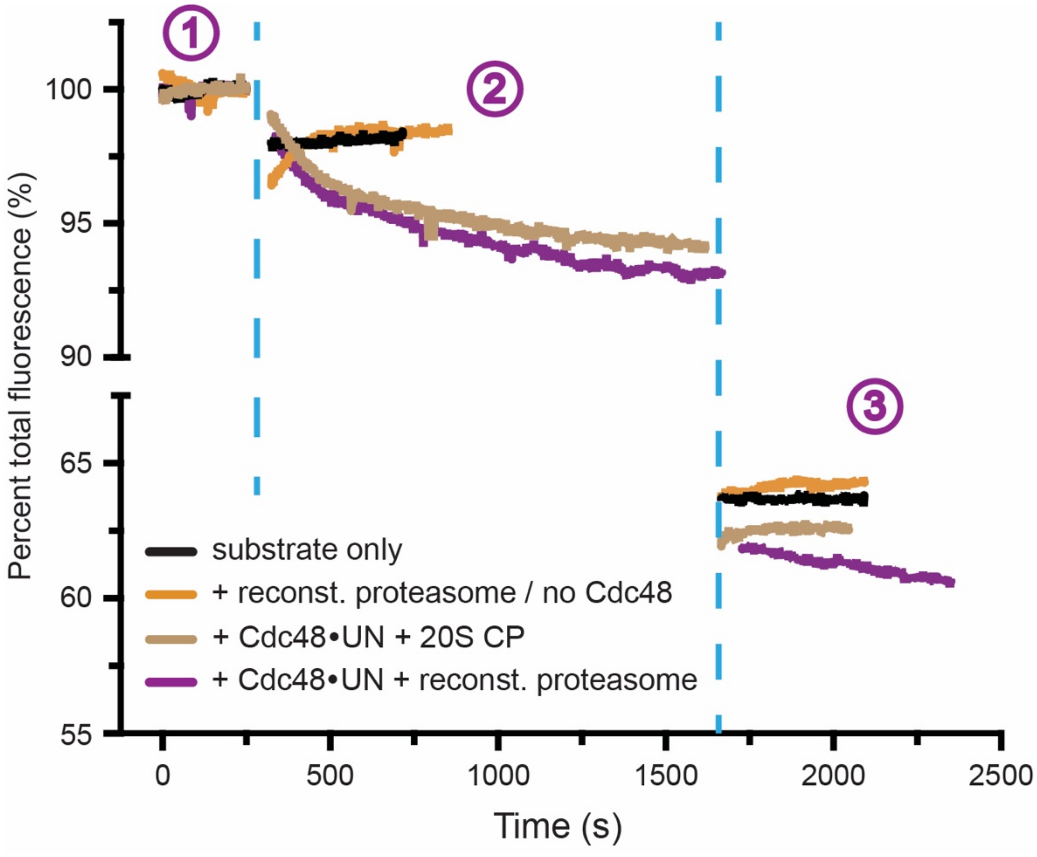

Supplementary Figure 4. Controls for Cdc48-mediated substrate degradation by the 26S proteasome. Shown are normalized example fluorescence time courses for the processing of K48-GREEN by various combinations of Cdc48•UN, the 20S CP, and the *in vitro* reconstituted 26S proteasome. Similar to the experiments shown in Figure 2A, the substrate was equilibrated (1), before addition of Cdc48•UN led to unfolding (2). After reaching steady state, 20S CP or reconstituted 26S proteasome was added (3). Degradation of K48-GREEN after unfolding by Cdc48•UN was not supported by isolated 20S CP (tan trace), but required the additional inclusion of the 19S regulatory particle to yield 26S proteasome holoenzymes (purple trace). The same reconstituted proteasome was unable to degrade K48-GREEN in the absence of Cdc48•UN (orange trace).

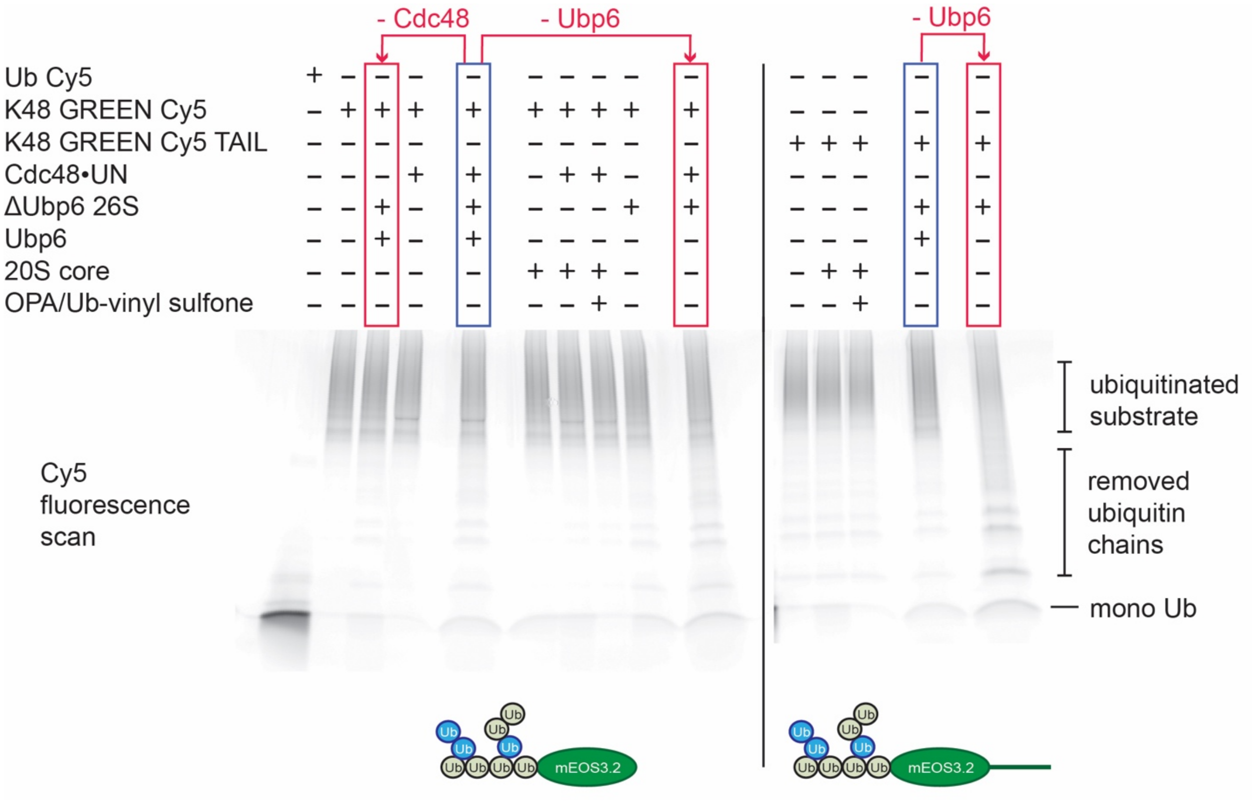

Supplementary Figure 5. Degradation-coupled substrate deubiquitination at the proteasome after unfolding by Cdc48. K48-GREEN and K48-GREEN-TAIL were modified with a mix of Cy5-labeled and unlabeled ubiquitin, and subjected to processing by various combinations of Cdc48, isolated 20S CP, and 26S proteasome in the absence or presence of Ubp6 and inhibitors of deubiquitination (o-phenanthroline/OPA and ubiquitin-vinyl sulfone). The reaction end points were analyzed by SDS-PAGE and Cy5-fluorescence detection. Robust deubiquitination was observed for K48-GREEN in the presence the 26S proteasome and Cdc48•UN, as well as for K48-GREEN-TAIL with 26S proteasome alone. Degradation-coupled deubiquitination was partially inhibited by the presence of Ubp6 on the proteasome. The tailed and untailed substrates showed identical deubiquitination patterns.

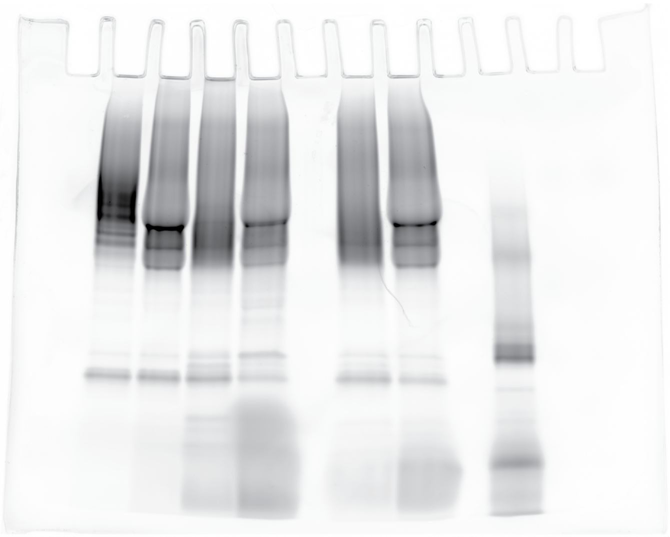

Supplementary Figure 6. Image of entire gel from Fig. 2C.

Plasmid name	Plasmid description
pAM104	His6-PreScissionSite-Cdc48 in pETduet
pAM105	Ufd1-PrescissionSite-His6 in pET24a(+)
pAM106	Npl4 in pETduet
pAM107	His6-PreScissionSite-Ufd2 in pETduet
pAM108	His6-Thrombin-SUMO-Ub(4)-mEOS3.2-intein-Chitin Binding Domain in pET15b
pAM109	His6-Thrombin-SUMO-Ub(4)-mEOS3.2-cyclin tail- -intein-Chitin Binding Domain in pET15b

Supplementary Table 1. Constructs used for the recombinant expression of Cdc48, Ufd1, Ufd2, Npl4, and the untailed and tailed mEOS3.2 model substrates with linear tetra-ubiquitin fusions.

	k (1/minute)	k2 (1/minute)	A1/A2
K48 GREEN non-activated	0.59 +/- 0.06		
K48 GREEN activated	0.56 +/- 0.1		
K48 RED	0.72 +/- 0.02	0.19 +/- 0.01	1.83 +/- 0.46

Supplementary Table 2. Rate constants and amplitude ratios for the unfolding of K48-GREEN and K48-RED by Cdc48•UN under single-turnover conditions, as derived from single- and double-exponential fits shown in Figure 1D-F (N = 3, repeats with identical protein samples).
