# Supplementary material for: The Cdc48 unfoldase prepares well-folded protein substrates for degradation by the 26S proteasome
Source: Commun Biol. 2019 Jan 21;2:29. doi: 10.1038/s42003-019-0283-z (PMC6340886; doi:10.1038/s42003-019-0283-z)
Supplement: Supplementary file 3 — Description of Additional Supplementary Files [file 42003_2019_283_MOESM3_ESM.docx]

**Description of Additional Supplementary Files**

**File Name**: Supplementary Data 1

**Description**: Raw data related to figures 1 and 2
